# Identification of Absorption Spectrum for IED Precursors Using Laser Photoacoustic Spectroscopy

**DOI:** 10.3390/molecules28196908

**Published:** 2023-10-02

**Authors:** Ana-Maria Bratu, Mioara Petrus, Cristina Popa

**Affiliations:** National Institute for Laser, Plasma and Radiation Physics, 409 Atomistilor St., P.O. Box MG-36, Magurele, 077125 Bucharest, Romania; ana.magureanu@inflpr.ro (A.-M.B.); mioara.petrus@inflpr.ro (M.P.)

**Keywords:** CO_2_ laser, photoacoustic spectroscopy, explosives-related molecules identification

## Abstract

Among the many commonly encountered hazards, improvised explosive devices (IEDs) remain the primary threat to military and civilian personnel due to the ease of their production and the widespread availability of their raw materials and precursors. Identifying traces of potential precursors is the first step in developing appropriate control measures. An interesting approach is to identify the precursors that are released around the site as they are handled and transformed into the final IEDs. CO_2_ laser photoacoustic spectroscopy can offer the spectral characterization of a number of explosives-related compounds without sample preparation. Benzene, toluene, acetone, and ethylene glycol absorption spectra were determined in the IR region between 9.2 and 10.8 µm. Each substance emitted a unique photoacoustic response corresponding to its chemical composition that could be further used to identify the explosive material.

## 1. Introduction

Over the last decade, the illicit use of explosive materials has intensified, particularly for the manufacture of improvised explosive devices (IEDs). Their use to carry out terrorist bombings continues to pose a serious threat to the safety and security of the entire world. Unrestricted access to the necessary technical information directed the IED producers to manufacture the explosives or explosive mixtures in clandestine laboratories [1]. These aspects have led to the study of ways to prevent the manufacture of explosives from commercially available chemicals [2,3,4,5]. Thus, banning or restricting access to some chemical substances, tracking their commercial circuits, obtaining information on the necessary techniques and technology, locations, or suspicious persons have been strong assets for combating the manufacture of explosives and have increased the effectiveness of the possible response from the authorized forces.

The chemical substances used for the production of explosives are not classified as explosive materials but may include chemical compounds or elements that, through various reactions or a series of chemical reactions that are relatively easy to achieve, can be transformed into explosive substances [6]. Many of the chemicals in question are currently used in the chemical industry. In addition to the manufacture of explosives, propellants, and powders, they are used in the manufacture of varnishes and paints, glass and ceramics, in the pharmaceutical, fertilizer and insecticide industries, in food additives or substances necessary for the processing of agricultural products, rubber, natural leather and cellulosic materials, cooling agents, fuels, etc. Limiting the access to some chemicals is a difficult task, which would create disruptions to the retail industry and inconvenience for peaceful consumers or small industrialists. 

Explosives and explosives-related substance identification and detection is still under development because a lot of physical-chemistry issues are involved in this very challenging area. Laser-based spectroscopy methods, due to their high sensitivity, selectivity, and multicomponent detection, created new solutions for identifying and characterizing explosive materials [7]. Raman spectroscopy [8], Fourier transform infrared spectroscopy [9], laser-induced breakdown spectroscopy [10], terahertz spectroscopy [11], gas chromatography-mass spectrometry [12], and ion mobility spectrometry [13] are commonly used methods for explosives identification and detection. Although it is used less than the other methods, laser photoacoustic spectroscopy presented important results in the detection of hazardous materials by using a quantum cascade laser and CO_2_ lasers [14,15]. Laser photoacoustic spectroscopy (LPAS) has several advantages including operator safety, non-destructive inspection, high sensitivity and selectivity, real time data analysis, immunity to electromagnetic interferences, no need for sample preparation, good time response, and a large dynamic range to measure low concentrations. LPAS is a very promising technique for the detection and identification of trace gases especially when a CO_2_ laser is used as the radiation source. The capabilities of the CO_2_ laser to be tunable and to emit a high output power make them ideal sources to characterize explosives-related substances. Even though CO_2_ lasers offer limited tunability in the range of 9–11 µm, many substances show strong absorption bands in the MIR region. The photoacoustic response also depends on the absorption coefficient at CO_2_ laser wavelengths of each explosives-related substance.

This study focuses on the identification of explosive chemical agents using CO_2_ LPAS. Common commercial explosive materials such as benzene, toluene, acetone, and ethylene glycol were analyzed as IED precursors. The absorption for various CO_2_ laser transitions was measured and the photoacoustic response of the tested substances was determined using spectrum analysis.

## 2. Results

CO_2_ LPAS is used for the identification of explosives-related substances. In order to start the process of determining the absorption spectra of the target substances, the PA cell must be cleaned so that there are no gases that can interfere with the sample gas. This process was realized with alternating nitrogen and vacuum flushing procedures until the background signal in nitrogen was around 35 µV. The absorption spectrum of the air was then identified, with the blotting paper in the enclosure having a reference role in determining the absorption spectra of the samples at the wavelengths of the CO_2_ laser. Each gaseous sample was obtained by completely evaporating 0.1 mL of the standard solution at 25 °C inside the 1.5 L volume glass enclosure together with room air and was introduced into the PA cell. The CO_2_ laser was tuned and directed onto the sample and the PA signal of the sample was recorded for each laser emission. The same procedure was adopted for each compound and the absorption spectra were investigated.

**Figure 1:**
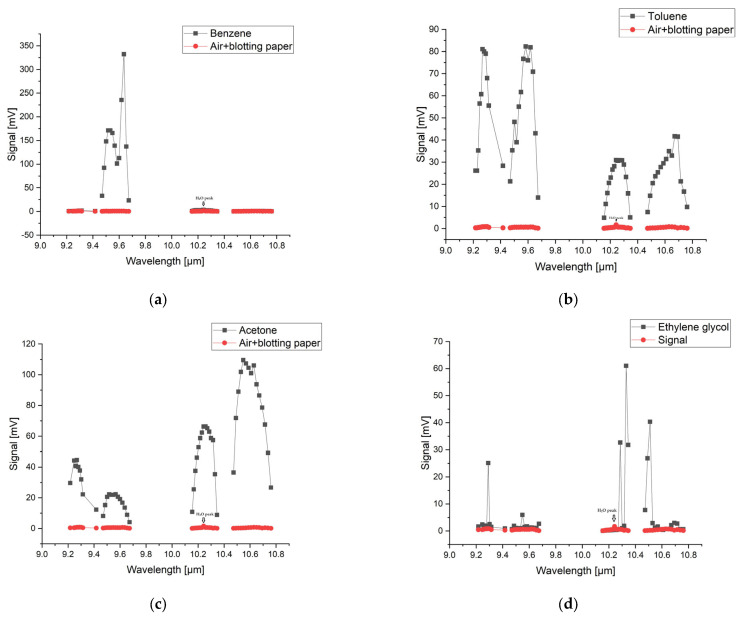
The absorption spectra for (**a**) benzene, (**b**) toluene, (**c**) acetone and (**d**) ethylene glycol in the 9.2–10.8 µm range on the 9P, 9R, 10P, and 10R branches of the CO_2_ laser.

Figure 1 shows both the reference spectrum and the absorption spectra for benzene, toluene, acetone, and ethylene glycol based on the signal measurements in the 9.2–10.8 µm range in the gas form compared with the spectra of the ambient air and blotting paper at atmospheric pressure. The figure also shows the absorption peak of water from the ambient air. The water peak is also marked on the graphs.

The reference signal shows in the 10R branch a small increase due to the presence of water vapor, but it is very small compared to the sample signals. The laser power inside the cell during spectral acquisition was between 0.5 W on the weaker lines and 3 W on the stronger lines. Each substance has its own chemical properties and has a characteristic IR spectral signature and its own absorption lines. The maximum absorption of benzene was observed in the 9P branch at the 9.636 µm wavelength of the CO_2_ laser, corresponding to the 9P(30) laser line. Toluene shows several absorption peaks, especially in the 9 µm range with four signal maxima at the wavelengths 9.583 µm, 9.618 µm, 9.258 µm, and 9.268 µm, corresponding to the lines 9P(24), 9P(28), 9R(22), and 9R(20).While benzene and toluene have a similar chemical structure and they absorb in the 9 µm region, acetone and ethylene glycol show absorption in the 10 µm region. For acetone, the 10P(28) and 10P(20) lines at 10.671 µm and 10.588 µm in the 10P branch are highlighted. Ethylene glycol presents a maximum signal value on the 10R(8) line at 10.330 µm and two smaller values on the 10R(6) lines at 10.346 µm and 10R(14) at 10.286 µm. By determining the absorption spectra of these substances, their maximum absorption was observed in the range 9.2–10.8 µm. Knowing these absorption lines, target compounds can be easily detected quantitatively.

## 3. Discussion

CO_2_ LPAS proved to be a suitable method for the identification of substances related to explosives by determining their characteristic absorption lines in the MIR region. The method presents advantages in the identification of explosives such as: (a) a very small amount of sample; (b) there is no pre-treatment of the sample (inside a glass cuvette that was filled with ambient air solution, each solution sample was evaporated and then moved into the PA cell. All measurements were made at atmospheric pressure, and the sample in the PA cell was a mixture of ambient air and vapors resulting from evaporation from each individual solution. The evaporation rate was practically obtained in a few hours); (c) no destruction of the sample during analysis; (d) measurements made in air at atmospheric pressure; (e) low cost per operation. The ability of CO_2_ laser photoacoustic spectroscopy to characterize and identify explosives was demonstrated by Patel in 2008 [16] and by Giubileo et al. in 2010 [17]. Following the analysis of the measured PA signals of the substances benzene, toluene, acetone, and ethylene glycol using CO_2_LPAS (Figure 1) it can be seen that they present measurable absorptions in the 9.2–10.8 µm range. This is not surprising since the tuning range of the laser falls within the MIR, which is known to be the fingerprint region where the significant absorption bands of organic molecules fall. Considering that benzene, toluene, acetone, and ethylene glycol are organic compounds, the strong absorption lines detected can be attributed mainly to the absorption bands of the movements of the C-H group, present in explosive chemical agents. From the point of view of explosives identification, it can be seen in Figure 1 that the PA signals obtained for each analyzed sample present a different distribution of absorption peaks, a different spectral behavior which facilitates the unequivocal identification of the species using CO_2_ LPAS. The identification of substances at known concentrations was carried out in the spectral range 9.2–10.8 μm. By measuring the PA signal at the wavelength of the maximum absorption case it gives us the opportunity to determine the absorption coefficient of the target species. Knowing the absorption coefficient and the wavelength from which the species has maximum absorption, we can determine the concentrations in the range of ppb to ppm from a mixture of gases. A glass vessel with a volume of 1.5 L filled with ambient air and one of four substances, i.e., acetone, ethylene glycol, benzene, and toluene, was used for these measurements. The approximate concentration values for the absorption signals using the CO_2_LPAS system were 112 ppm for benzene, 94 ppm for toluene, 135 ppm for acetone, and 99 ppm for ethylene glycol.

**Figure 2:**
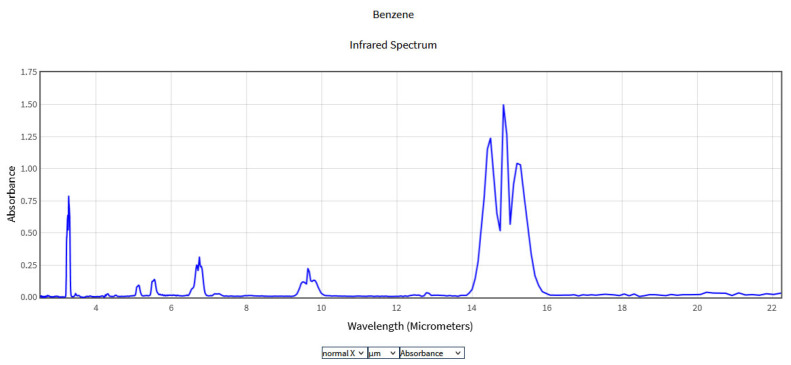
IR Spectrum of Benzene in the NIST Mass Spectrometry Data Center [20].

**Figure 3:**
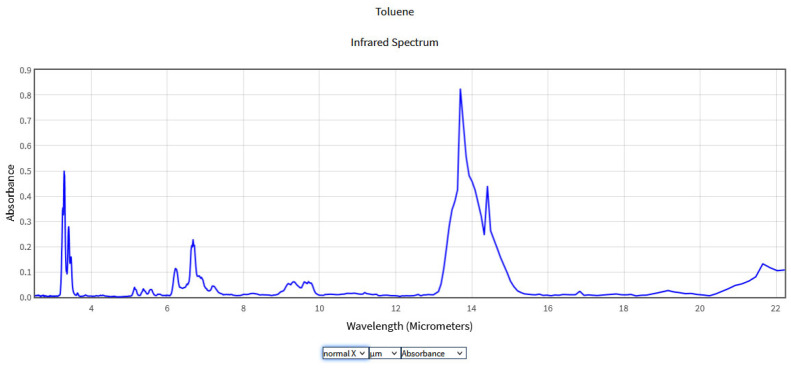
IR Spectrum of Toluene in the NIST Mass Spectrometry Data Center [21].

**Figure 4:**
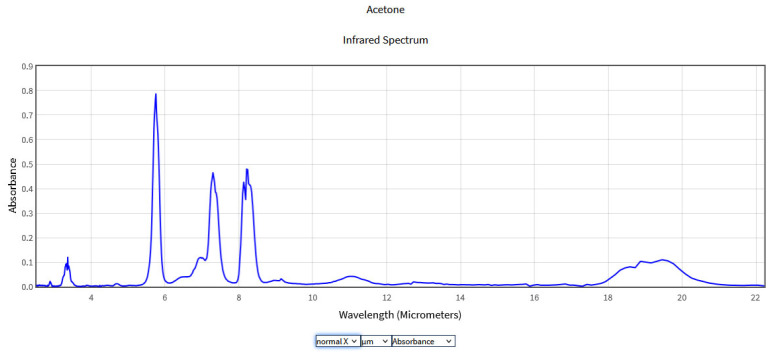
IR Spectrum of Acetone in the NIST Mass Spectrometry Data Center [25].

**Figure 5:**
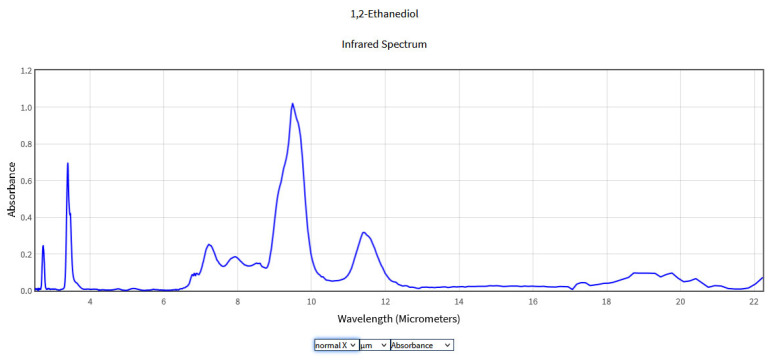
IR Spectrum of Ethylene glycol in the NIST Mass Spectrometry Data Center [27].

The obtained spectra were compared with the National Institute of Standards and Technology (NIST) database (Figure 2, Figure 3, Figure 4 and Figure 5), which provides quantitative and qualitative data on infrared spectra for standard gases. The main absorption peaks associated with aromatic hydrocarbons are presented by NIST mass spectrometry [18]. In the spectral region between 2 and 22 μm, the IR spectra of benzene and toluene are characterized by several absorption bands, the most prominent due to the vibrations of the C–H bonds and C=C double bonds in the molecule, but there are also bands of additional absorption due to vibrations of other functional groups in the molecule. The IR spectra of benzene showed four prominent peaks: the peak between 3.12 and 3.57 μm that represents one or more C–H stretches, the peak between 6.17 and 7.14 μm that is attributed to C=C stretching frequencies of aromatic groups, the peak between 8.33–10 μm that is caused by in-plane C–H bend mode, and the peak between 10 and 14.28 μm that indicates the out-of-plane C–H bend modes. The weak peaks between 5 and 6 μm are a series of overtone and combination bands called the benzene fingers. For toluene, the infrared absorption is as for a benzene compound with a single methyl group (CH_3_) substituent in the benzene ring (CH_3_). The extra peaks between 3.35 and 3.50 μm are due to a symmetric and antisymmetric CH_3_ stretch. The peaks between 6.80 μm and 7.30 μm correspond to a CH_3_ antisymmetric and symmetric bend [19].

In the 8.3 and 10.52 μm range, a similarity can be seen between the absorption spectra of benzene and toluene obtained using CO_2_LPAS and those obtained using mass spectrometry presented by NIST due to the aromatic C-H in plane bands [22]. Although gas chromatography and mass spectrometry offer a greater spectrum of identification for NIST, these devices often require periodic gas sampling and are also very expensive. In the nearby area, at 9.77 µm, the benzene spectrum was investigated by J.Waschull et al. using tunable diode laser spectroscopy [23]. Ihor Sydoryk et al. analyzed the absorption features of benzene and toluene between 9.88 and 9.41 μm at atmospheric pressure using a tunable continuous-wave external cavity quantum cascade laser [24]. 

As can be seen from Figure 4 the IR spectrum of acetone presented four prominent peaks at 3–4 μm, 5.5–6 μm, 7–7.5 μm, and 8–8.5 μm. The peak around 3–4 μm corresponds to the C-H stretching motion of the methyl group. In the interval 5.5–6 μm, the absorption peak is attributed to the C=O stretching motion of the carbonyl group. The peaks from the interval 7–7.5 μm and 8–8.5 μm are attributed to the C-C stretching motion of the carbonyl group and the C-H bending motion of the methyl group, respectively. The absorption peaks within the range of 10 μm are the attributes of various C-O stretches of acetone and will appear as a weaker peak. Because of weak absorption, the 10 μm region has been less studied. Studies in the 10 μm region were carried out by Mitrayana also with the help of a CO_2_ laser photoacoustic spectrometer, finding an absorption maximum at the wavelength of 10.59 m [26].

The typical IR spectrum of ethylene glycol presents an absorption at 2.98 μm corresponding to the O-H stretching mode and at 3.44 and 3.57 μm due to the stretching modes of the CH_2_ groups. The strong band in the range of 9–10 μm is associated with the C-O stretching modes. Groups occurring at 6.66–7.50 μm can be attributed to the unsaturated C-C bonds but were not identified on the basis of the tables of specific bonds. Ethylene glycol is mainly detected using gas chromatography and mass spectrometry [28]. IR signatures of the gaseous ethylene glycol were studied by Kira et al. using open-path FTIR obtaining spectral models in the 9–11 μm region [29].

Infrared techniques were also applied for differentiating the absorption spectra of DNT and TNT and other explosives or IED precursors [15,17,30,31,32].

Although rarely used as an integrated system for the on-line detection of trace explosives in the vapor state, CO_2_LPAS has been shown to be a rapid, sensitive, and selective tool for the determination of trace explosive chemical agents.

## 4. Materials and Methods

### 4.1. Materials

Starting from a relatively long list of explosive chemical agents, we focused our study on the 4 gaseous compounds benzene, toluene, acetone, and ethylene glycol analyzed with LPAS. The samples were procured from the chemistry laboratory of the National Institute of Lasers, Plasma and Radiation Physics. Benzene, toluene, acetone, and ethylene glycol are solutions used in the chemical industry that can form the basis of the manufacture of explosives or some pyrotechnic mixtures or compositions.

1. Benzene (C_6_H_6_)
  - Is a colorless liquid aromatic hydrocarbon, and is highly flammable. Benzene reacts with concentrated nitric acid in the presence of concentrated sulfuric acid at a temperature not exceeding 55 °C to give nitrobenzene, an intermediate for explosives [33].

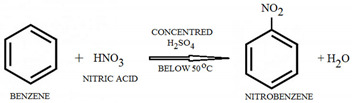

2. Toluene (C_7_H_8_)
  - Is also a liquid aromatic hydrocarbon which in the nitration reaction reacts 25 times faster than benzene. Toluene is first nitrated with a solution of sulfuric acid and nitric acid and mononitrotoluene is created. After being separated, the mononitrotoluene is used to create dinitrotoluene. In the last phase, trinitrotoluene is obtained by nitration of dinitrotoluen using an anhydrous solution of nitric acid and oleum [34].

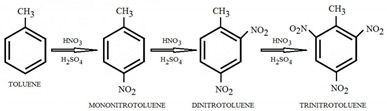

3. Acetone (C_3_H_6_O)
  - Is the simplest ketone. Using the commonly available household chemicals acetone and hydrogen peroxide, it is easy to synthesize a powerful explosive such as triacetone triperoxide (TATP). TAPT is obtained by mixing acetone with concentrated 30–50% hydrogen peroxide solution in an acidic environment (typically sulfuric acid). TAPT is the most used IED because of its readily available and inexpensive precursor materials coupled with its widely available instructions [35].

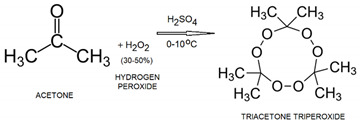

4. Ethylene glycol (C_2_H_6_O_2_)
  - Is a clear, colorless, odorless liquid, soluble in most organic solvents. Through nitration of ethylene glycol, ethylene glycol dinitrate can be obtained, which belongs to the category of nitrate ester liquid explosives and can be initiated through mechanical stimuli, friction, or heat [36].

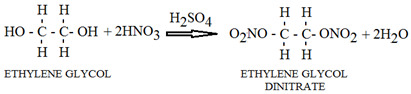



Ethylene glycol dinitrate is sometimes mixed with nitroglycerin in several kinds of dynamite to reduce its freezing temperature [37]. 

From each solution, 0.1 mL were extracted with a syringe, which were then transferred to a 2 × 8 cm absorbent paper that includes a glass support (microscope slide) and placed in a stick vat where they were allowed to evaporate for 3 h at a temperature of 25 °C. Evaporation was carried out under normal atmospheric conditions to simulate real conditions. 

### 4.2. Method

Identification of the explosive chemicals benzene, toluene, acetone, and ethylene glycol were performed using CO_2_LPAS. The entire spectroscopic system consists of a CO_2_ laser and a photoacoustic (PA) cell in which the gas is detected and analyzed in combination with a gas handling system [38,39,40]. 

**Figure 6:**
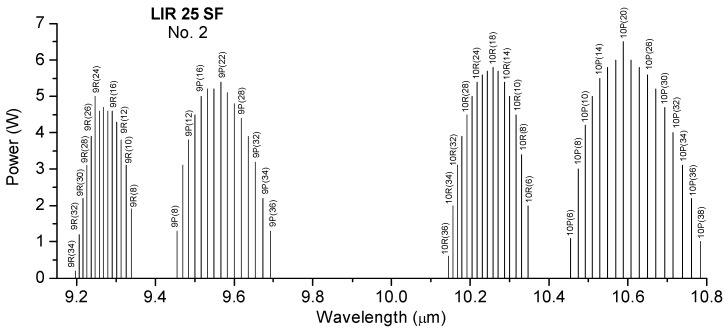
CO_2_ laser emission spectrum.

The CO_2_ laser is frequency-stabilized, emitting in continuous wave (cw), and is tunable in the spectral range of 9.2–10.8 µm on 54 distinct spectral lines as a result of vibrational–rotational transitions grouped in the 4 branches 9R, 9P, 10R, and 10P. The tunability of the CO_2_ laser is shown in Figure 6.

The laser power varies between 0.5 and 6.5 W depending on the emitted laser transition. The photoacoustic spectrum is realized by tuning the laser to different wavelengths and measuring the pressure changes in the cell. Via this spectrum, the maximum absorption of the sample can be identified. The characteristics of the laser significantly influence the kind and number of detectable substances.

The resonant photoacoustic cell is 300 mm long and 7 mm in diameter. The acoustic resonator works at 564 Hz and is characterized by:

- Quality factor Q = 16.1, calculated as the ratio between the resonance frequency and the frequency band, at 1/√2 of the maximum signal amplitudes;
- Cell constant C = 4375 Pa·cm/W which describes the sensitivity of the PA resonator at a given resonance frequency;
- The response capacity of the PA cell R = 350 cmV/W, calculated as the product of the cell constant and the sensitivity of the microphones.

The values of the cell parameters cell constant C and responsivity R were measured experimentally with calibration measurements using certified gas mixtures.

To obtain the experimental value of the cell constant C, a calibrated mixture of 1 ppm of C_2_H_4_ in N_2_ at a total pressure of 1 atm was used. The measurements were made on the 10P(14) line of the CO_2_ laser, where α = 30.4 cm^−1^atm^−1^. While the sensitivity of S_M_ microphones is constant over the frequency range used, the cell constant C is proportional to the measured photoacoustic signal: V = αCS_M_P_L_c.

For c = 10^−6^ atm, α = 30.4 cm^−1^atm^−1^ and S_M_ = 80 mV/Pa, the cell constant C = 4375 Pa·cm/W was obtained.

The responsivity of the cell, R [V × cm/W], is defined as the amplitude of the electrical signal delivered by the microphones per unit of power absorbed by the molecules per unit length: R = C·S_M_.

For the above data, the responsivity of the cell is R = 350 V·cm/W. The photoacoustic signal V, measured as the output of the microphones becomes V = αRP_L_c.

**Figure 7:**
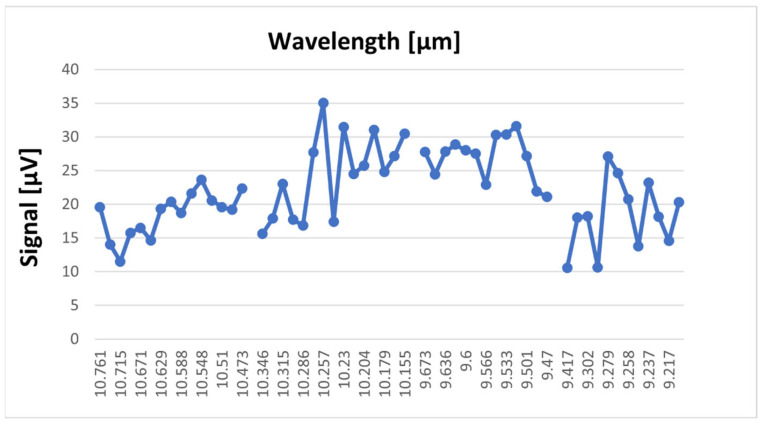
The absorption spectra of CO_2_ laser radiation in pure nitrogen 6.0.

Before sample measurements, a background absorption spectrum of pure nitrogen was made in order to estimate the signal-to-noise ratio (see Figure 7).

The maximum signal in nitrogen was about 35 µV and was considered the photoacoustic background signal.

Another important part of the LPAS system is the gas handling system, which is constructed of flexible PFA Teflon tubes (PFA-T6M-1M-30M, 6 mm), valves (SS-1GS6), and junction TEs (SS-6M0-3, brand Swagelok), to clean the PA cell, introduce the sample gas into the PA cell at a controlled flow rate, evacuate the sample gas from the cell, and monitor the pressure of the gas mixtures. 

Once the gas is introduced into the PA cell, the absorption spectrum at all laser wavelengths can be measured. The resulting PA signal for each laser line depends on the absorption of the gas at that wavelength. In this process, the continuous CO_2_ laser radiation is amplitude-modulated by a mechanical modulator operating at the same frequency as the PA cell and then focused by a ZnSe lens and directed to the PA cell. Inside the cell, the target gas molecules selectively absorb a small amount of radiation being excited on the vibrational–rotational level. The non-radiative deactivation of the excited molecules produces local heating and a pressure variation, generating acoustic waves in the resonator. Acoustic waves produced in the PA cell are detected by four miniature microphones connected in series. In photoacoustic measurements in the gas phase, microphones are usually employed as sensing elements of the acoustic waves generated by the heat deposition of the absorbing molecules. Although high-quality condenser microphones offer the best noise performance, they are rarely used in photoacoustic gas detection because of their large size, lower robustness, and relatively high price. The most common microphones employed are miniature electret devices originally developed as hearing aids. The choice of a miniature microphone is particularly advantageous since it can be readily incorporated in the resonant cavity without significantly degrading the quality factor Q of the resonance. In our PA cells there are four Knowles electret EK-3033 or EK-23024 miniature microphones in series (sensitivity 20 mV/Pa each at 560 Hz) mounted flush with the wall. They are situated at the loops of the standing wave pattern, at an angle of 90° to one another. The microphones are coupled to the resonator by holes (1 mm diameter) positioned on the central perimeter of the resonator. The battery-powered microphones are mounted in a Teflon ring pulled over the resonator tube. It is of significant importance to prevent gas leakage from inside the resonator tube along the Teflon microphone holder, since minute spacing between the holder and resonator tube produces a dramatic decrease of the microphone signal and the Q value. The electrical output from these microphones is summed and the signal is selectively amplified by a two-phase lock-in amplifier tuned to the chopper frequency.

**Figure 8:**
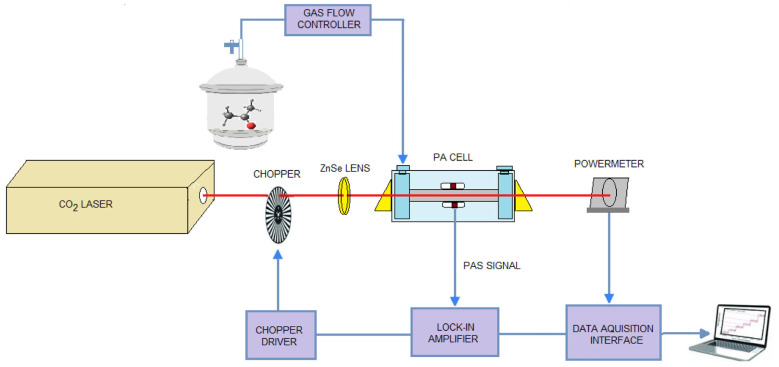
Scheme of the experimental laser photoacoustic spectroscopy system.

The microphones convert the acoustic signal into an electrical signal which is then fed into a dual-phase lock-in amplifier, synchronized with the frequency of the modulator. We used a dual-phase, digital lock-in amplifier Stanford Research Systems model SR 830 with the following characteristics: full scale sensitivity, 2 nV–1V; input noise, 6 nV (rms)/Hz at 1 kHz; dynamic reserve, greater than 100 dB; frequency range, 1 mHz–102 kHz; time constants, 10 μs–30 s (reference > 200 Hz), or up to 30,000 s (reference < 200 Hz). The power of the laser beam is considered constant in the PA cell, so the power read at the exit of the cell by the radiometer represents the real value. The PA signal and laser power at the cell exit are automatically recorded in real time by a National Instruments acquisition board (NI cDAQ-9174) and transmitted to the computer(see Figure 8).

## 5. Conclusions

The identification of explosive chemical agents in gaseous form was achieved by determining the absorption spectra using CO_2_LPAS. The concept of recognizing chemical agents leading to the formation of explosive precursors was demonstrated by performing measurements on the selected set of chemicals. Absorption spectra for benzene, toluene, acetone, and ethylene glycol in the range 9.2–10.8 µm were obtained and compared with NIST spectra. The maximum values of the signals were observed on the 9P(30) line for benzene; on the 9P(24), 9P(28), 9R(22), and 9R(20) lines for toluene; the 10P(28) and 10P(20) lines for acetone; and the 10R(6) line for ethylene glycol. It was observed that the PA signals obtained for each analyzed sample present a different distribution of absorption peaks; a different spectral behavior which facilitates an unequivocal identification of the species. The extremely high sensitivity of this technique allows for the practical development of procedures for the early detection of traces of explosives. This study will have important implications for current studies trying to monitor explosives and their precursors, as well as for future studies trying to develop low-cost models for the rapid identification and detection of explosives.

## Data Availability

Data supporting the findings are available from the corresponding authors upon reasonable request.

## Sample Availability

Samples are available.
